# Tracheal agenesis: the importance of teamwork in an uncommon pathology, challenging diagnosis, and high mortality—a case report

**DOI:** 10.3389/fped.2024.1401729

**Published:** 2024-07-11

**Authors:** Belén Fernández Monteagudo, Salvador Piris Borregas, Lidia Niño Díaz, Tania Carbayo Jiménez, Rocío Morante Valverde, Jesús Vicente Redondo Sedano, Maria Teresa Moral Pumarega

**Affiliations:** ^1^ Servicio de Neonatología, Hospital Universitario 12 de Octubre, Madrid, Spain; ^2^ Pediatric Surgery Unit, University Hospital October 12, Madrid, Spain

**Keywords:** tracheal agenesis, respiratory distress, tracheoesophageal fistula, single umbilical artery, polyhydramnios

## Abstract

**Introduction:**

We present the case of a patient with an unexpected postnatal diagnosis of tracheal agenesis, a severe and rare pathology with fewer than 200 cases documented in the literature, typically diagnosed postmortem. In our instance, early diagnosis was achieved through collaborative efforts and teamwork among various professionals. We provide illustrative images and videos to assist colleagues in identifying this congenital anomaly.

**Case presentation:**

The patient is a term newborn with prenatal indicators of polyhydramnios and a single umbilical artery. Upon birth, the infant exhibited severe respiratory distress, and orotracheal intubation via direct laryngoscopy was unfeasible. Consequently, an urgent fibrobronchoscopy, conducted by pediatric surgeons, led to the diagnosis of tracheal agenesis with tracheoesophageal fistula and the placement of a directed endotracheal tube. This intervention facilitated temporary ventilation until parental consensus on management was achieved. Following a multidisciplinary consultation, the decision was made to proceed with extracorporeal membrane oxygenation. Unfortunately, the patient experienced a prolonged refractory cardiorespiratory arrest and died after 7 h of life in his mother’s arms.

**Conclusion:**

Teamwork in neonatology is indispensable when addressing emergent pathologies. In our experience, multidisciplinary management, including anesthesiologists and pediatric surgeons, should be contemplated in complex scenarios.

## Introduction

Tracheal agenesis is a congenital airway anomaly first described in 1900 by Payne et al. (1, 2). It is a rare disease, with an incidence of 1 per 50,000–100,000 newborns (3) and only 174 cases internationally described in the literature (4). The prevalence is high in males, with a 2:1 ratio (5). It has been associated with some antenatal features such as polyhydramnios and intrauterine growth retardation, some affected newborns are born prematurely (1, 2). The disease also has a high mortality rate. Only 10 patients are known to survive at 12 months of life, most of whom require several surgical procedures. None have survived beyond 10 years (4).

From a clinical point of view, the congenital disease is characterized by partial or complete absence of the trachea (3). In some cases, there is a communication between the gastrointestinal tract and the tracheoesophageal tube, known as a bronchoesophageal fistula, which allows for provisional and partial ventilation of these patients postnatally (5–7).

Currently, the etiology of tracheal agenesis is unknown. It is believed to occur around day 26 of intrauterine life during embryogenesis of the respiratory diverticulum, depending on the primitive intestine. The respiratory tract develops as a diverticulum from the ventral region of the anterior intestine, which lengthens caudally to form the trachea and the rest of the bronchial structures. The development of the tracheoesophageal ridge, which will become a septum separating the trachea and the esophagus, is also crucial. Tracheal agenesis occurs when none of the above processes takes place (7).

**Figure 1:**
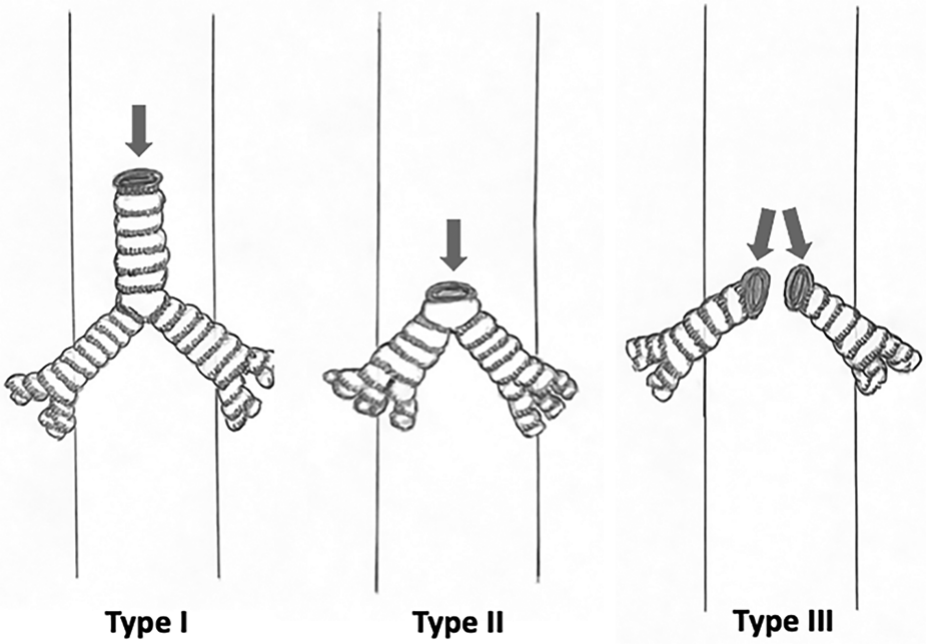
Diagrams showing Floyd's classification.

The most commonly used classification for tracheal agenesis is the classification of Floyd et al. in 1962 (see Figure 1) (8, 9). It describes three types of tracheal agenesis depending on the degree of development and the type of connection with the esophagus (3, 5). Type I presents agenesis of the proximal trachea and a short distal tracheal remnant with a tracheoesophageal fistula. Type II is complete agenesis of the trachea with bronchi communicating with the carina through a tracheoesophageal fistula. Finally, type III is complete agenesis of the trachea, and the bronchi communicate through separate fistulas with the distal esophagus. Type II is the most frequent, with a frequency of 60%, followed by types I and III, with a frequency of 20% (5). A more complete classification was proposed by Faro et al. (10), which includes seven types. We can highlight Type A, which implies total pulmonary agenesis, and types F and G, in which there is no tracheoesophageal fistula. However, some cases do not fit into any of these categories (9).

Tracheal agenesis may appear alone or in association with other congenital anomalies. In most cases, the associated anomalies are cardiovascular, including septal defects, valvular defects, transposition of great arteries, or tetralogy of Fallot. Genitourinary and gastrointestinal anomalies and umbilical vessel alterations are also frequent in these patients (1, 2, 11, 12). In addition, it can be associated with syndromes such as CHARGE association (13) (coloboma, heart anomaly, choanal atresia, retardation of growth, and genital and ear anomalies), VACTERL (vertebral defects, anal atresia, radial dysplasia, renal defects, and cardiovascular defects) (4, 9), TACRD (tracheal agenesis/atresia, congenital heart disease, radial defects, and duodenal atresia), or FRASER (cryptophthalmia, syndactyly, laryngeal and tracheal anomalies, and urogenital malformations) (1, 2). This pathology can go unnoticed in prenatal diagnosis where a tracheoesophageal fistula exists, as the airway secretions flow through the digestive tract (6). However, some prenatal findings can alert clinicians, such as the presence of polyhydramnios, intrauterine growth retardation, and alterations in the umbilical vessels (5, 6). On the other hand, in types A (total pulmonary agenesis), F, and G (absence of tracheoesophageal fistula), prenatal diagnosis can be easier due to the absence of pulmonary tissue in the first case and the resemblance to congenital upper airway obstruction syndrome (CHAOS syndrome) in the remaining cases (14, 15). In these cases, enlarged lungs, flattened diaphragms, polyhydramnios, and hydrops fetalis are typical findings. If suspicion arises, fetal magnetic resonance imaging can help confirm the diagnosis (15, 16).

Postnatal diagnosis of tracheal agenesis is an emergency (17) and should be suspected in a newborn presenting with severe respiratory distress, inaudible crying, inadequate air entry on pulmonary auscultation, and the inability to perform endotracheal intubation by expert professionals (1). It should be noted that it is recommended not to force the entry of the endotracheal tube to avoid perforation of the airway structures. The urgent participation of a surgical team to perform fibrobronchoscopy is critical in the diagnosis (see Supplementary Video S1). In addition, the performance of tracheostomy is also a critical diagnostic step, considering that no tracheal lumen can be seen during the surgical procedure. A multidetector CT allows us to quickly obtain three-dimensional images and to detail the anatomy of the airway and tracheoesophageal fistula. It is useful not only for early diagnosis but also for planning surgical treatment (18).

The management of this congenital disease should involve a multidisciplinary team, including neonatologists, pediatric anesthesiologists, and pediatric surgeons. The initial priority of the team is to keep the airway patent and ventilate the patient through the esophagus via the tracheoesophageal fistula. Usually, an endotracheal tube is placed in a guided manner through a fibrobronchoscope (17) (see Supplementary Image S1). In cases of clinical deterioration or lack of improvement, the option of extracorporeal membrane oxygenation (ECMO) (1) should be considered, in consultation with the family (7). Although surgery remains the curative or definitive treatment option, very few patients have been treated surgically due to high mortality rates within the first 48 h of life; hence, there is not enough experience or no standardized surgical treatment protocol available (16).

We present our experience with a case of tracheal agenesis. We would like to highlight the multidisciplinary management and the family participation during the decision-making process.

## Patient and clinical information

Our patient was a 39 + 6-week male newborn with a birth weight of 2,940 g (13th percentile, −1 SD). As background, his parents were from Morocco. The mother was 28 years old and had six gestations and four miscarriages in the first trimester. She had a healthy 2-year-old son.

The pregnancy serologies were negative, except for rubella, which was indeterminate, and cytomegalovirus, which was IgG-positive and IgM-negative. The cervical exudate culture for *Streptococcus agalactiae* was negative.

The pregnancy was monitored at a high-risk gynecology center due to gestational diabetes treated with diet, persistent elevation of resistance in uterine arteries, single umbilical artery, a persistent right umbilical vein in its intrahepatic variant, and mild to moderate polyhydramnios diagnosed in the third-trimester ultrasound. There were no other structural anomalies, and the parents did not desire a genetic study. The water bag broke four and a half hours before delivery, with clear fluid, without fever or other infectious symptoms.

The birth took place by vaginal delivery. The newborn did not show effective respiratory effort, prompting vigorous stimulation and ventilation with intermittent positive pressure. Despite this, he continued without respiratory effort and with a heart rate below 100 beats per minute; then, orotracheal intubation was performed with a 3.5-cm Endotracheal tube (ETT), which was mistakenly positioned at 12 cm from the commissure, a distance longer than the standard depth for the patient's size. Following these maneuvers, the heart rate increased above 100 lpm, and the preductal peripheral oxygen saturation (SaO_2_) achieved 90%. He was transferred to the neonatal intensive care unit (NICU) requiring 100% oxygen without any incidence.

On physical examination, no dysmorphism or dyschromia were found. The lip, palate, and clavicles appeared intact, and the anus was permeable and normally positioned. Lung auscultation revealed symmetrical ventilation with abundant respiratory secretion noises. In the NICU, the patient became increasingly vigorous, so we decided to extubate him on non-invasive ventilation. Immediately, he developed severe respiratory distress characterized by generalized cyanosis, absence of audible crying, and severe hypoventilation on venous gasometer analysis. Reintubation was performed by the neonatology team under sedation, during which the supraglottis and glottis were visualized as normal, and the ETT passed through the vocal cords. Nevertheless, despite five attempts by expert neonatologists, the ETT could not be passed beyond the vocal cords. In this context, we requested help from the anesthesiology team, who attempted it six more times, but intubation could not be attempted. Throughout this process, we alerted the pediatric surgical team, who performed a fibrobronchoscopy and confirmed the diagnosis.

## Diagnostic assessment

Fibrobronchoscopy (see Supplementary Video S1) revealed a normal supraglottis and glottis but with an obstruction at the subglottic level. Initially, it was interpreted as a probable congenital subglottic membrane or stenosis, so an emergency tracheostomy was performed. In this procedure, we observed a lack of airway continuity beyond the cricoid cartilage. We performed immediate esophagoscopy and visualized a fistula that ended in a primitive and non-functional structure resembling a tracheal carina (see Supplementary Video S2). As with other complementary tests, chest x-rays showed the absence of the tracheal pattern, poorly expanded lungs, and an apparently normal digestive system. X-rays of the spine and extremities were normal. The urgent echocardiogram showed an aberrant right aortic arch, a left subclavian artery, and an open ductus arteriosus with a bidirectional shunt. Blood tests including complete blood count and liver and kidney function were normal. We found persistent respiratory acidosis in the blood gases. According to the general parental visitation policy of our NICU, the father was allowed continued presence. Every step of the diagnostic process and the findings were communicated progressively to him. The clinical relevance and severity of the pathology were emphasized, as well as the probability of death. Because of the language barrier, we used diagrams and images to improve comprehension.

## Treatment, follow-up, and outcome

As an emergent treatment, an ETT guided by esophagoscopy was inserted through the esophagus via the tracheoesophageal fistula and placed in the tracheobronchial remnant, 14 cm from the commissure. Therapeutic management was urgently discussed in a multidisciplinary session between the neonatology team, surgery, and the patient's family. The patient required increased respiratory support to high-frequency ventilation with a Sensor Medics instrument, reaching maximum parameters of mean pressure of 18 cmH_2_O, amplitude of 40 cmH_2_O, frequency of 8 Hz, and FiO_2_ persistently at 100%. Initially, the patient maintained peripheral SaO_2_ around 90%–92% with this respiratory support, but severe hypoventilation persisted with a pH below 7.20 and pCO_2_ between 80 and 100 mmHg. In this context, urgent ECMO was assessed, and the patient's family was informed. The severity and poor prognosis of the disease were emphasized, with a high possibility of death despite treatment. The parents opted for active management, and the ECMO protocol of our unit was proposed. Nevertheless, the condition of the patient worsened, experiencing greater hypoxemia, persistent inability to achieve adequate ventilation, and hypotension. Finally, the patient suffered a cardiorespiratory arrest, which could not be reversed despite several doses of adrenaline. Due to the deterioration, lack of response to the measures taken, and poor prognosis, he died after 7 h of life in his mother's arms. The parents did not authorize an autopsy, but a chest x-ray with barium contrast was performed (see Supplementary Image S2).

Prior to decease, a blood sample was obtained for whole-exome sequencing, revealing no genetic variants that could explain the clinical presentation. However, the pathogenic variant c.1066-11T>G in the *PAH* gene associated with autosomal recessive classical phenylketonuria was detected in heterozygosity. This information was communicated to the family.

## Timeline

**Figure 2:**
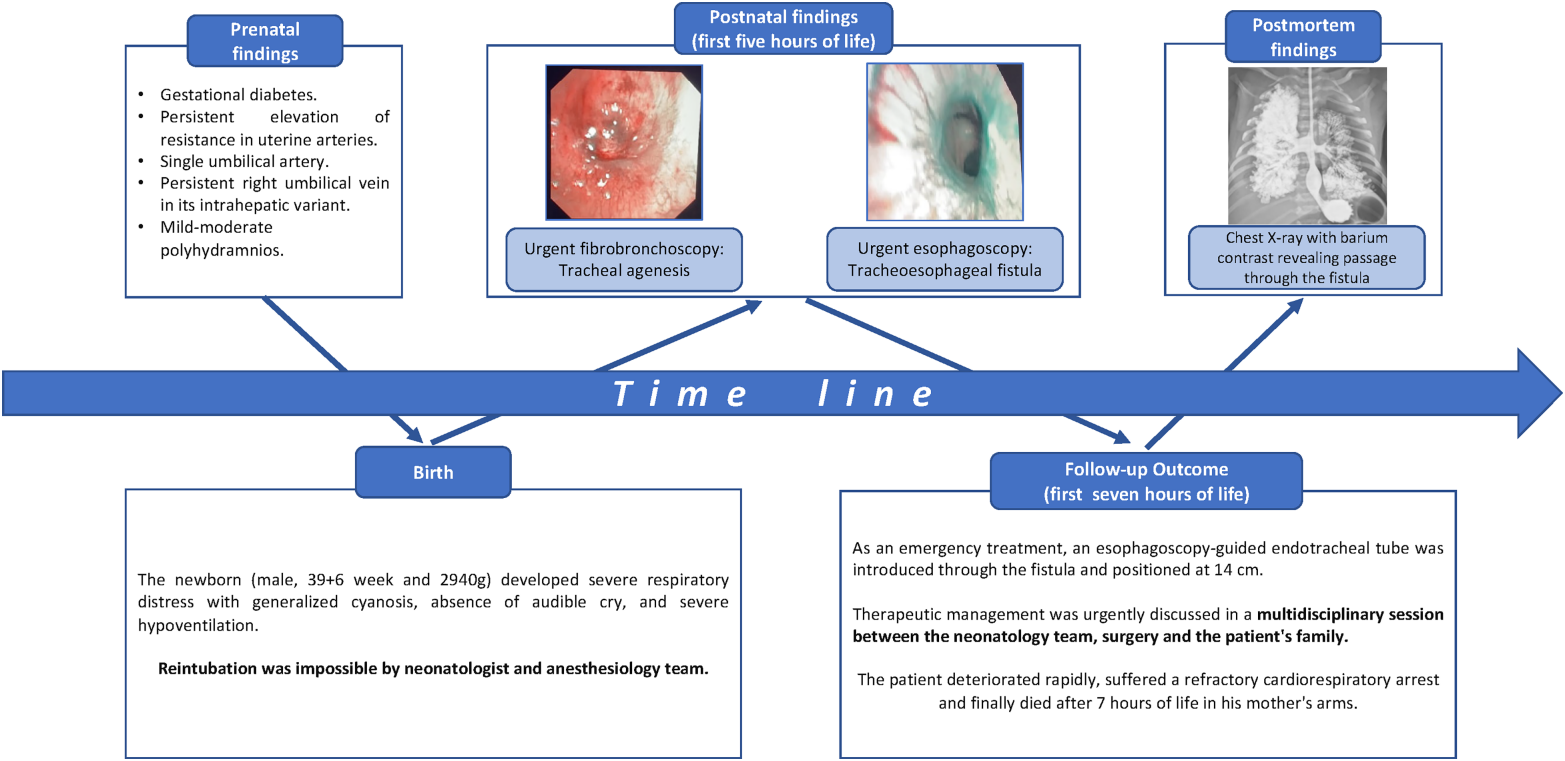
Diagram showing a time line from the patient's birth.

Details of timeline are present in Figure 2.

## Discussion

We present the management of a case involving an extremely rare pathology with high mortality. The prenatal diagnosis is extremely complicated; therefore, the postnatal diagnosis is unexpected for neonatologists and depends on a high degree of suspicion. Unfortunately, most diagnoses are made postmortem, and for those made premortem, obtaining high-quality images that alert the neonatal team to early diagnostic signs is unusual. When these signs are recognized, management is quicker and more direct. It allows us to manage the communication process with the family, involving them in decision-making and, in the event of the patient's death, facilitating the mourning process.

Tracheal agenesis is a very rare pathology (1, 2), with very few cases described in the literature and an estimated mortality rate of around 85%. Most patients die in the first 48 h of life due to respiratory failure. The diagnosis is achieved postmortem in most cases (16).

Survival in cases of tracheal agenesis depends on airway management and the possibility of selective intubation of the fistula through the esophagus (17). In fact, in a recently published article by Wu et al., regarding three cases of tracheal agenesis, when faced with a patient clinically suggestive of tracheal agenesis, immediate esophageal intubation should be attempted to ventilate the patient through the tracheoesophageal fistula as a life support measure (19). In our case, unintentional intubation in the esophagus was performed in the delivery room, and survival for a few hours was possible thanks to teamwork with anesthesia and surgical teams to maintain a patent airway. It is necessary to highlight the importance of avoiding forceful intubation to prevent rupture of the airway structures. Also, effective communication with the family is essential once the diagnosis and initial survival management have been established (7). Especially if the family has not been present in the diagnostic process, and if possible, all the professionals should be involved. In our case, showing diagrams or drawings to parents of Moroccan origin with language barriers helped us to understand each other. If the parents opt for active management, ECMO therapy can serve as a bridge to decide on subsequent treatment (1).

In 2000, Crombleholme et al. (20) published a case report detailing a patient prenatally diagnosed with CHAOS syndrome due to tracheal atresia, in which the EXIT technique was successfully performed, ensuring a patent airway at birth. Subsequently, at 17 months of age, tracheal reconstruction utilizing costal cartilages was performed. While our case differs in that our patient presented with tracheal agenesis and that the diagnosis was postnatal; this EXIT technique holds potential utility in rare instances of prenatal diagnosis or strong suspicion of tracheal agenesis.

Currently, there is no standardized surgery for this pathology due to limited experience and fewer patients. However, based on the series of patients with tracheal agenesis reported by Straughan (4), in almost all of them, palliative surgery was performed as an intermediate step to definitive treatment, consisting of distal esophageal ligation, creating an esophageal airway, proximal esophagostomy to divert salivary secretion, and gastrostomy for enteral feeding. In the second stage, reconstruction of the transit with a graft, usually colonic, could be performed (17, 21). These palliative interventions have two important problems. Esophageal tract collapsibility can lead to frequent hospitalization in the context of respiratory failure. In addition, the possibility of accidental decannulation can appear, as occurred in the case reported by Straughan, in which the patient finally died (4). To avoid these complications, internal or external esophageal supports have been proposed to achieve tracheal rigidity (22, 23), ranging from internal polytetrafluoroethylene rings (22) to external resorbable splint-type supports made of polycaprolactone. The last one was used in 2021 by Tsai et al., with encouraging results because the patient was discharged at 13 months of life without respiratory support (22). Another alternative for the management of these patients is tracheal transplantation, which is currently under investigation (9).

In our case, given the rapid deterioration of the patient and subsequent death, surgical measures were not considered. The team's intention, guided by the family's wishes, was to continue treatment and initiate ECMO support. Therefore, a limitation in this case was the restricted therapeutic management employed based fundamentally on life support measures. There is a possibility that earlier initiation of ECMO support could have led to a different outcome for the patient, but the mortality rate among these patients remains exceedingly high. For this reason, before commencing ECMO, comprehensive information was provided to the family through a multidisciplinary approach, allowing them to decide whether to continue or discontinue treatment.

As a reinforcement, we consider that the diagnosis was made promptly, and the parents were actively involved in decision-making after receiving information from all the professionals involved.

We believe that our case report may help other professionals, as we provide a report of the diagnostic process with images and two video reports of fibrobronchoscopy and esophagoscopy. We also like to highlight the importance of the multidisciplinary team in a difficult situation and the participation of the parents during the entire decision-making process.

## Data Availability

The original contributions presented in the study are included in the article/**Supplementary Material**, further inquiries can be directed to the corresponding author.

## Supplementary material

Supplementary Image S1 Chest radiograph showing that the ETT is positioned lower than normal. It was positioned under guided esophagoscopy assistance through the tracheoesophageal fistula to enable ventilation.

Supplementary Image S2 Radiograph taken postmortem. The contrast was administered to the patient via an orogastric tube. Observe the connection between the digestive tract and the respiratory tract.

Supplementary Video S2 Fibroscopy performed by pediatric surgery. Observe a normal supraglottis and glottis but with a stop at the subglottic level.

Supplementary Video S2 Esophagoscopy performed by pediatric surgery. Observe a tracheoesophageal fistula that ended in a primitive structure similar to a tracheal carina.
