# Supplementary figures and images for: Tracheal agenesis: the importance of teamwork in an uncommon pathology, challenging diagnosis, and high mortality—a case report
Source: Front Pediatr. 2024 Jul 11;12:1401729. doi: 10.3389/fped.2024.1401729 (PMC11269142; doi:10.3389/fped.2024.1401729)

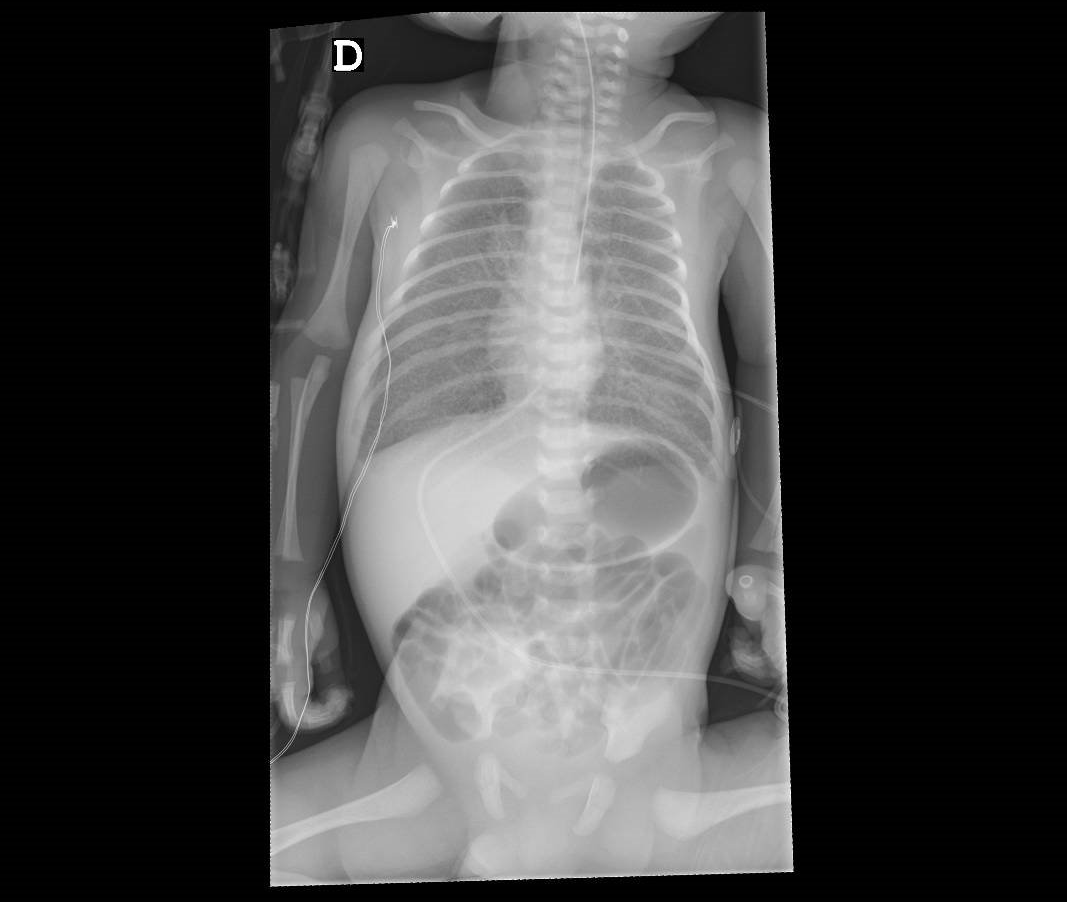

Supplement: Supplementary Image S1 — Chest radiograph showing that the ETT is positioned lower than normal. It was positioned under guided esophagoscopy assistance through the tracheoesophageal fistula to enable ventilation. [file Image1.png]

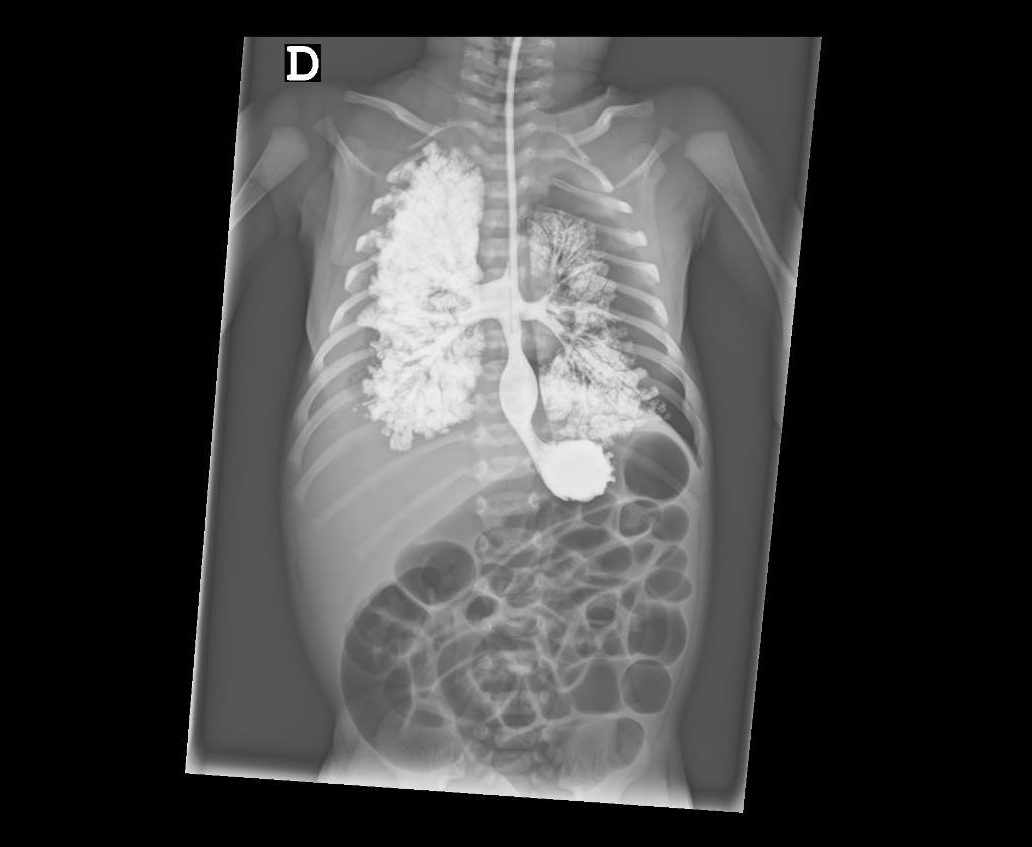

Supplement: Supplementary Image S2 — Radiograph taken postmortem. The contrast was administered to the patient via an orogastric tube. Observe the connection between the digestive tract and the respiratory tract. [file Image2.png]
